# Long term cost outcomes among commercially insured patients undergoing bariatric surgical procedures

**DOI:** 10.1002/osp4.727

**Published:** 2024-01-01

**Authors:** Sonali Shambhu, Qinli Ma, Aliza S. Gordon, David Pryor, Joseph A. Karam, Andrea DeVries

**Affiliations:** ^1^ Public Policy Institute Elevance Health Indianapolis Indiana USA; ^2^ Enterprise Health Service Research Elevance Health Wilmington Delaware USA; ^3^ Clinical Strategy and Innovation Elevance Health Woodland Hills California USA; ^4^ Grievances & Appeals Elevance Health Woodland Hills California USA

**Keywords:** bariatric surgery, health economics, health services research, obesity

## Abstract

**Objective:**

Bariatric procedures have become safer in recent years, warranting new data on long‐term costs. This study examined the impact of bariatric procedures on a person's long‐term healthcare costs up to 10 years and if it differed by socio‐economic status (SES).

**Methods:**

This retrospective observational study compared the downstream health care cost of patients with obesity who had undergone bariatric surgery (BS) between 2009 and 2018 to a 1:1 matched group of members with obesity but no surgery.

**Results:**

167,764 individuals from administrative claims data with an obesity diagnosis were included; 83,882 in the BS group and 83,882 in the non‐surgical group. In follow‐up years 2–10, the BS group was associated with lower total medical healthcare cost compared to the non‐surgical group (cost ratios ranged 0.85–0.93, *p* values < 0.05). When stratifying the BS group by SES quartiles, there were no significant cost differences by SES (cost ratios ranged from 0.96 to 1.05, most *p* values > 0.05).

**Conclusions:**

BS was associated with lower long‐term follow‐up medical cost and cost savings appeared similar among the SES quartiles in the BS group. The study results may help policy makers and employers in designing benefits and extending coverage for bariatric surgical procedures.

## Graphical abstract

In our study, with 10‐year follow‐up data, we find that bariatric surgery (BS) is associated with lower follow‐up cost from Year 2 to Year 9 compared with a matched cohort. We believe that the lower cost in the surgery group is mainly due to newer, safer bariatric surgeries and lower medication spending. This study can provide guidance to policy makers, researchers, and clinicians to make informed decisions regarding long‐term cost benefits associated with BS. It can also help payors and employers understand the benefits of covering BS.

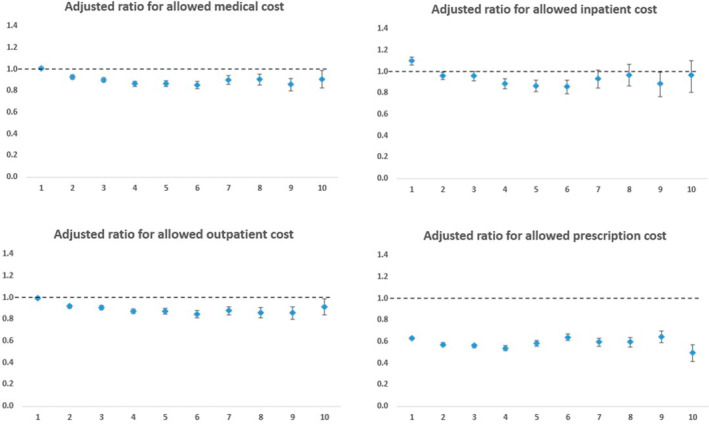

## 1 INTRODUCTION

From 1999 to 2020, the prevalence of obesity in the United States (US) increased from 30.5% to 41.9% and severe obesity (defined as Body Mass Index [BMI] ≥ 40 kg/m^2^) rose from 4.7% to 9.2%.^1^ The increase occurred across all income and education levels. It is known that low neighborhood‐level socio‐economic status (SES) is associated with a higher risk of obesity.^1^, ^2^, ^3^, ^4^

Severe obesity has become a significant health burden for individuals and the US health care system, with 1 in every 11 US adults suffering from it. According to a recent study, severe obesity accounted for $126 billion in 2019 medical spending.^5^ Average annual medical costs for adults with obesity and severe obesity exceeded those of their peers who did not have obesity by $1900 and $3100, respectively. Most excess costs were associated with the treatment of obesity‐related diseases such as diabetes, coronary heart disease and hypertension.^5^ While the economic and social costs of severe obesity are well‐documented, there are few effective options available that result in sustained weight loss.

Currently, the most effective option available is bariatric surgery (BS), which became increasingly popular in the late 1990s. Existing research has demonstrated superior effects of BS on weight loss and glycemic control compared to traditional medical approaches for treating obesity.^6^, ^7^, ^8^, ^9^ In addition, laparoscopic approaches have made BS safer and less invasive in the past decade^10^, ^11^ and common bariatric procedures have also changed over time, with the Sleeve Gastrectomy (SG) gaining popularity in recent years.^11^, ^12^, ^13^ Studies show that the SG is comparably more effective than Adjustable Gastric Band (AGB) and Roux‐en‐Y Gastric Bypass (RYGB) (five‐year mean total weight loss of 25.5% for RYGB, 18.8% for SG, and 11.7% for AGB)^12^ and 30‐day composite adverse events were less common following SG (2.8%) than other bariatric surgeries (RYGB [3.8%] and AGB [3.1%]).^11^

Even though bariatric surgeries have evolved and are becoming increasingly common, there are racial and socioeconomic status disparities in the use of BS.^3^, ^4^, ^14^ A recent study of the use of BS and SES status observed an inverse linear relationship between BS rates and SES status among the white population, and for racial minorities, the surgery rates were lower in the lowest SES quintiles.^14^ A number of factors have been identified that may account for the difference in the use of the procedure, including differences in patient and physician views and attitudes, patient‐physician communication, and concerns about the cost of the procedure.^15^ While a difference in the relationship between SES status and the use of the procedure has been well‐documented, little is known about the relationship between SES status and long‐term post‐BS outcomes. One study of BS outcomes after surgery between Medicaid and non‐Medicaid patients found that weight loss was similar between the two groups at a median follow‐up of 3.1 years and that social determinants of health at the neighborhood level and Medicaid status were not associated with weight loss outcomes after BS.^16^

In addition to clinical effectiveness, data on healthcare cost associated with BS are important for designing health benefit coverage, given that it is an elective procedure. One study using health plan data from 2002 to 2008 showed that the BS cohort did not experience cost savings in the 6 years post‐surgery relative to a matched control group.^17^, ^18^ Given the recent improvements in clinical outcomes for available procedures and the lack of current cost information on these procedures, new economic evaluations of bariatric surgeries are warranted.

The objective of this study was to estimate the impact of bariatric procedures on health care costs measured up to 10 years after surgery. In addition, longitudinal costs were stratified by SES status in the BS group to determine the role of SES on long‐term cost outcomes among patients who underwent BS. More research is needed to better understand their post‐surgery outcomes because people with lower SES status may have a harder time managing the new diet and lifestyle requirements as they might be more likely to live in deprived neighborhoods with increased poverty, food insecurity and poor access to exercise and post‐surgery care.^3^, ^19^, ^20^

## 2 METHODS

### 2.1 Surgical group identification

**FIGURE 1:**
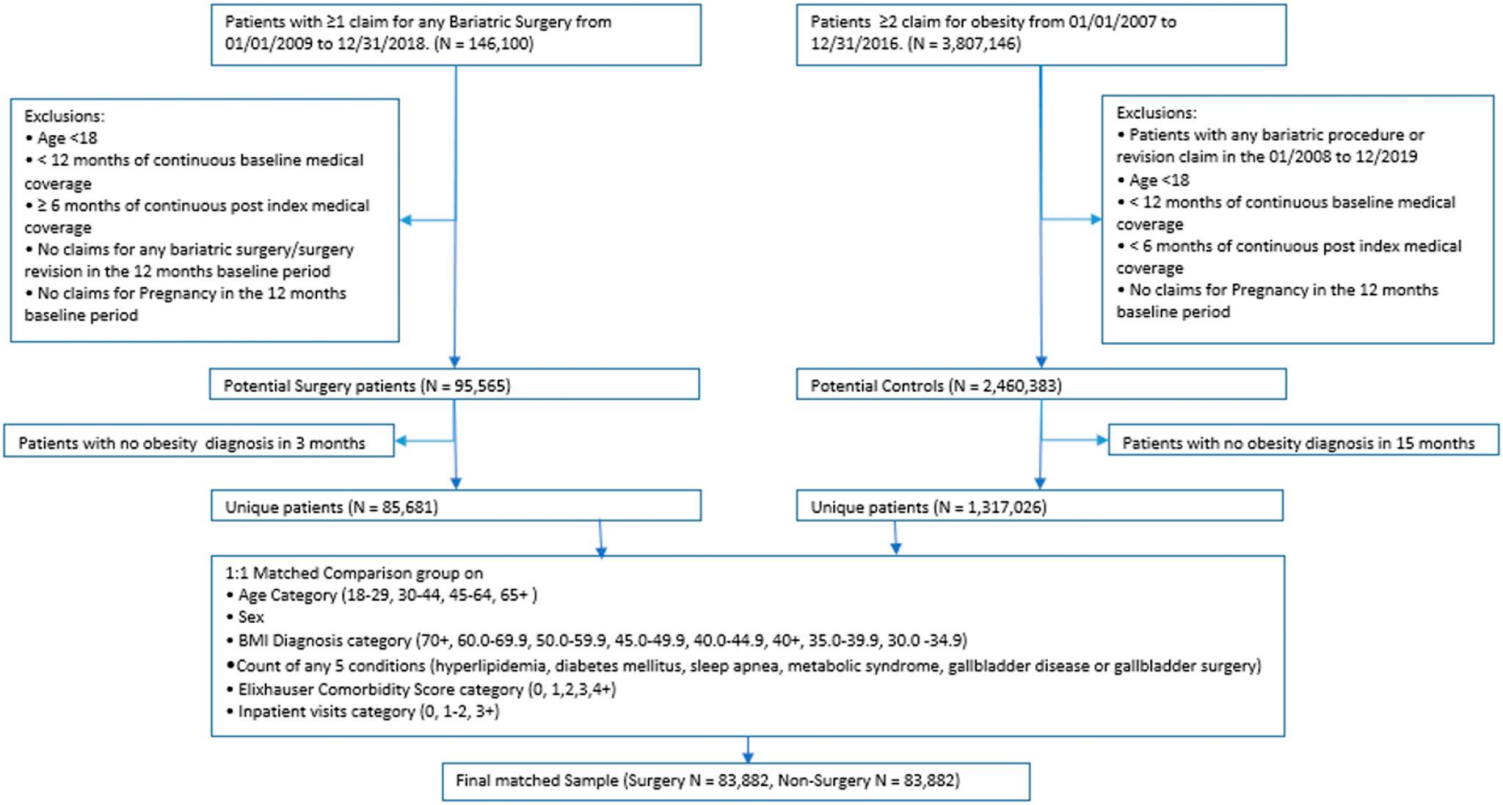
Study sample attrition.

This retrospective observational study used administrative claims data to compare medically insured enrollees in commercial insurance plans who underwent BS between January 1, 2009 and December 31, 2018 to a matched group with an obesity diagnosis who did not undergo BS. Among patients who underwent surgery, surgical episodes were created using an index date defined as the date of the BS. The enrollees were required to be adults (aged ≥18) with 12 months pre‐index and 6 months post‐index continuous enrollment. For all post‐operative periods after year 1, study participants enrolled in their health plans for more than 1 day that year were included in cost measure calculations. Enrollees with any prior BS, surgery revision, or pregnancy claims during the 12‐month pre‐index period were excluded. (see Figure 1)

### 2.2 Comparison group identification

#### 2.2.1 Candidate pool for the comparison group

**FIGURE 2:**
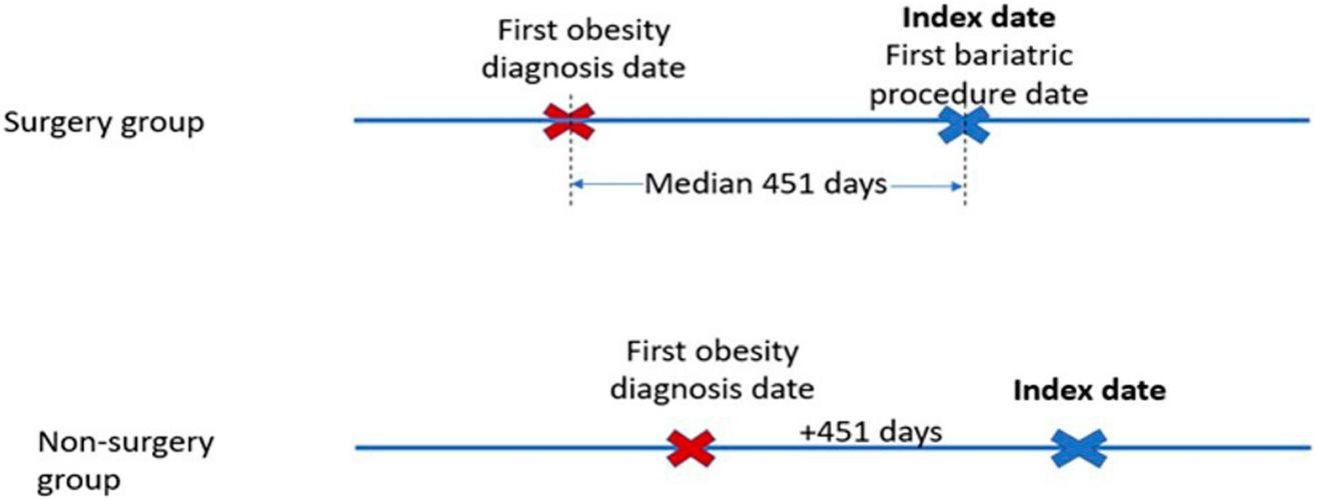
Index date assignment for the non‐surgical group.

The initial candidate pool for the non‐surgical comparison group included commercial health plan enrollees with at least two claims with an obesity diagnosis and no prior bariatric procedures during the study period. This cohort was assigned an index date 451 days after the first obesity diagnosis to mimic the index date of BS in the surgical group. The 451 days reflected the median time interval between the first obesity diagnosis and the BS date in the surgical group (see Figure 2). Both surgical and non‐surgical groups were identified using the Healthcare Intergraded Research Database (HIRD),^21^ a repository of longitudinal medical and pharmacy claims data of commercially insured individuals with health plans in 14 states in the US. All claims in the repository are final adjudicated claims.

#### 2.2.2 Comparison group matching

After applying the same inclusion/exclusion criteria as the surgical group, exact 1:1 matching was performed to identify a non‐surgical comparison group similar to the surgical group based on a variety of demographic and clinical characteristics in the 12‐month baseline period. The matching variables, based on Weiner et al.,^17^ were selected to ensure a balance of important characteristics associated with obesity and BS and to prevent pruning of observations.

The characteristics used for exact matching included eight BMI diagnostic categories (see Table S1), age category (18–29, 30–44, 45–64, 65+), gender, a composite count variable of five common obesity‐related chronic conditions (hyperlipidemia, diabetes mellitus, sleep apnea, metabolic syndrome, gallbladder disease or gallbladder surgery), Elixhauser comorbidity score categories (0, 1, 2, 3, 4+), and number of all‐cause inpatient (INP) admissions in the baseline period (see Table 2 [Exact matched variables]). It is noteworthy that BMI information is better documented in recent claims data. First, there are additional BMI categories in the ICD‐10 codes, allowing for a more precise match on weight, and second, BMI ICD 10 codes are more likely to be populated in claims data in recent years.

### 2.3 Study outcomes

The main outcome of interest was post‐index total medical costs (sum of INP, emergency room [ER] and outpatient [OUT] cost) during a follow‐up period that lasted as long as 10 years, depending on the duration of health plan enrollment following the index date. In addition to total medical costs, the subset of medical costs generated in the INP and OUT settings were analyzed separately. Furthermore, prescription costs were analyzed among the subset of the study population with available pharmacy data. All follow‐up costs were measured during 12 different time periods: the index surgery (operative) date, 30‐day post‐index, and annual post‐operative periods during the 10 years following the index date. The allowed amount variable from healthcare claims was used for all cost analyses.

### 2.4 Validation of claims‐documented BMI measures

Of note, BMI diagnosis codes were used to define obesity and match the surgical and non‐surgical groups. Validation of the ICD 9/ICD 10 diagnostic codes was performed with clinically measured BMI values available on a subset of the data. Among the study population, 3062 (3.7%) in the surgical group and 3906 (4.7%) in the non‐surgical group had clinically measured BMI values within 1 year prior to the index date. These BMI values were used to test and validate the diagnosis of high BMI in claims data. Among these claim‐based individuals with obesity, 95.0% in the surgical group and 87.9% in the non‐surgical group had BMI ≥ 35 kg/m^2^, indicating that BMI diagnosis codes are well coded in the claims data among the population.

### 2.5 SES analysis

A subgroup analysis was conducted on surgical patients to compare the cost differences following BS by SES categories. The surgical cohort was split into four quartiles of SES based on a composite score generated from seven variables from the 2018 American Community Survey, using the address of residence at the census block group level.^22^ Category one indicated the lowest SES. SES category three, the most common category in the study population, was used as the reference category.

### 2.6 Statistical analysis

Patient baseline characteristics were summarized as means with standard deviations for continuous variables and number and proportions for categorical variables. Standardized mean differences were calculated for surgical versus non‐surgical groups to check for covariate balance after exact matching. Multivariate generalized linear regression models (GLM) with a gamma distribution and log link were used to assess cost outcome measures.^23^ Note that since many baseline characteristics were measured but not included in the exact matching process, these characteristics were included as covariates in the GLM. Patients with zero costs during a study year were assigned a cost of $1 for model inclusion and high costs were capped at $250,000 to avoid results that were skewed by outlier cases. The assumption of a gamma distribution was tested with the modified Park test.^24^ Six months of continuous enrollment were required in the first post index year; for all other annual post‐operative periods (Year 2 to Year 10), study participants enrolled in a health plan for at least 1 day in the given 1‐year period were included in cost measure calculations for that year. Costs were inflation‐adjusted to the 2019 value and annualized to account for partial year plan enrollments.

All the multivariate statistical models were adjusted for both matched variables (to account for any differences in patient mix in follow‐up years due to attrition) and unmatched baseline covariates, including region, health plan type, index surgery type (only for SES subgroup analysis), procedure year, and demographic information obtained at the zip code level (i.e., race, ethnicity, median household income, SES, and education level). Baseline comorbidities like anxiety, depression, diabetes, eating disorder, thrombosis, dyslipidemia, gastroesophageal reflux disease, gall bladder surgery, metabolic syndrome, hypertension, chronic kidney disease, nonalcoholic fatty liver disease, osteoarthritis, and sleep apnea, as well as 12‐month baseline ER visits and 6–12 months baseline total cost were included in the model. A 6 month washout period for baseline total cost prior to the index was used to avoid adjusting for increasing cost in the surgery group due to pre‐surgical work‐up and evaluation. *E*‐values were calculated for all significant results to account for likely other unmeasured confounders that could account for differences in observed costs.^25^, ^26^ The *p*‐values for multiple SES quartile comparisons were adjusted by Dunnett–Hsu correction test. Statistical significance was set at a 2‐sided alpha = .05. A sensitivity analysis was conducted among members with 5 years continuous medical and pharmacy eligibility using a subset of pharmacy enrollment to ensure that members who left or enrolled back in a health plan were not substantially different in the two cohorts. All analyses were conducted using SAS Enterprise Guide version 7.15 (SAS Institute Inc).

### 2.7 Ethics

The study protocol was approved by the Regulatory Compliance team and Clinical Effectiveness Advisory Committee at Elevance Health, Inc. Informed consent was deemed unnecessary according to national laws and regulations, that is, the Health Insurance Portability and Accountability Act (HIPAA) and the HIPAA Privacy Rule (45 CFR 164.514(e)(3)i) since the study was conducted using a limited dataset for analysis devoid of individual patient identifiers. The study was initially conducted by and on behalf of the health plans quality improvement efforts concerning obesity management and post‐BS outcomes as a health care operation function pursuant to 45 CFR 164.506.

## 3 RESULTS

### 3.1 Study population

A total of 83,882 commercial health plan enrollees with obesity who had BS met the study criteria and were included in the surgical group. After matching, 83,882 enrollees with obesity who did not undergo BS were selected into the non‐surgical comparison group (shown in Figure 1). Table 1 includes the number of eligible patients in each group in each follow‐up period. The duration of post‐index date follow‐up is a function of disenrollment from health plans overtime. About two out of three patients were followed up for at least 3 years after the surgery and half were followed for at least 4 years following the surgery. The proportion of enrollees undergoing BS was lower (19.7%) in the lowest quartile (SES 1) of SES than in the other SES categories (see Table 2).

**TABLE 1:** Number of patients and percent remaining in the study group during 10 follow‐up periods.

Study group	Year 1	Year 2	Year 3	Year 4	Year 5	Year 6	Year 7	Year 8	Year 9	Year 10
Non‐surgical group	83,882 (100.0)	73,336 (87.4)	49,424 (58.9)	31,371 (37.4)	20,336 (24.2)	13,231 (15.8)	9169 (10.9)	6308 (7.5)	3934 (4.7)	2363 (2.8)
Surgical group	83,879 (100.0)	74,338 (88.6)	56,921 (67.9)	44,273 (52.8)	34,902 (41.6)	27,755 (33.1)	21,401 (25.5)	15,972 (19.0)	11,308 (13.5)	7084 (8.4)

**TABLE 2:** Baseline Characteristics of Surgical and Matched Non‐surgical Patients

Characteristic	Surgical group (*n* = 83,882)	Non‐surgical group (*n* = 83,882)	Standardized mean difference
Exact matched baseline characteristics			
Age category, no. (%)			
18–29	7192 (8.6)	7192 (8.6)	Exact matched
30–44	32,018 (38.2)	32,018 (38.2)	
45–64	41,254 (49.2)	41,254 (49.2)	
65+	3418 (4.1)	3418 (4.1)	
Gender, no. (%)			
Female	62,892 (75.0)	62,892 (75.0)	Exact matched
Male	20,990 (25.0)	20,990 (25.0)	
BMI diagnosis category, no. (%)			
BMI 70 and over	686 (0.8)	686 (0.8)	Exact matched
BMI 60.0–69.9	2557 (3.0)	2557 (3.0)	
BMI 50.0–59.9	11,949 (14.2)	11,949 (14.2)	
BMI 45.0–49.9	14,148 (16.9)	14,148 (16.9)	
BMI 40.0–44.9	22,069 (26.3)	22,069 (26.3)	
BMI 40 and over	32,111 (38.3)	32,111 (38.3)	
BMI 35.0–39.9	140 (0.2)	140 (0.2)	
BMI 30.0–34.9	222 (0.3)	222 (0.3)	
Baseline ECI category, no. (%)			
0	289 (0.3)	289 (0.3)	Exact matched
1	6297 (7.5)	6297 (7.5)	
2	15,072 (18.0)	15,072 (18.0)	
3	18,799 (22.4)	18,799 (22.4)	
4+	43,425 (51.8)	43,425 (51.8)	
Baseline utilization and cost			
Inpatient visit, no. (%)	6950 (8.3)	6950 (8.3)	Exact matched
Unmatched baseline characteristics			
Residence region, no. (%)			0.445
Northeast	19,058 (25.5)	10,783 (14.1)	
Midwest	12,911 (17.3)	20,388 (26.6)	
South	23,022 (30.8)	33,936 (44.3)	
West	19,660 (26.3)	11,422 (14.9)	
Ratio of race at zip code level, mean (SD)			
Black	0.1 (0.2)	0.1 (0.2)	−0.152
White	0.8 (0.2)	0.8 (0.3)	0.019
Asian	0.0 (0.1)	0.0 (0.1)	0.135
Hispanic	0.2 (0.2)	0.1 (0.2)	0.220
Socioeconomic status category, no. (%)			0.165
1 (most disadvantaged)	15,846 (19.7)	19,288 (23.9)	
2	19,882 (24.7)	22,815 (28.2)	
3	23,111 (28.8)	22,342 (27.6)	
4 (least disadvantaged)	21,516 (26.8)	16,405 (20.3)	
Baseline comorbidities, no. (%)			
Anxiety	18,969 (22.6)	18,072 (21.5)	0.026
Depression	27,446 (32.7)	21,646 (25.8)	0.152
Diabetes mellitus	28,882 (34.4)	39,574 (47.2)	−0.262
Thrombosis	2597 (3.1)	2919 (3.5)	−0.022
Eating disorder	4127 (4.9)	447 (0.5)	0.272
Hyperlipidemia	44,300 (52.8)	48,884 (58.3)	−0.110
Gastroesophageal reflux disease (GERD)	46,453 (55.4)	18,404 (21.9)	0.731
Hypertension	53,778 (64.1)	59,482 (70.9)	−0.146
Chronic kidney disease	7163 (8.5)	14,491 (17.3)	−0.263
Nonalcoholic steatohepatitis (NASH)	11,975 (14.3)	6854 (8.2)	0.194
Osteoarthritis	19,668 (23.4)	16,386 (19.5)	0.095
Sleep apnea	42,394 (50.5)	28,403 (33.9)	0.343
Baseline utilization and cost			
Emergency room visit, no. (%)	18,598 (22.2)	24,559 (29.3)	−0.163
Total cost, mean (SD)	$13,570 ($23,904)	$13,370 ($34,325)	0.007
6–12 months total cost, mean (SD)	$5405 ($14,444)	$6860 ($19,419)	−0.086

Abbreviations: BMI, body mass index; ECI, Elixhauser comorbidity index.

After matching, the surgical and non‐surgical groups had the same baseline profiles with respect to matched variables, as shown in Table 2. With respect to unmatched characteristics that were adjusted for in GLM, more surgery patients lived in the Northeast (25.5% vs. 14.4%) and West (26.3% vs. 14.9%) regions. Surgery patients were more likely to have eating disorder (4.9% vs. 0.5%), gastro‐esophageal reflux disease (55.4% vs. 21.9%), or sleep apnea (50.5% vs. 33.9%). Diabetes mellitus and chronic kidney disease were more common among non‐surgical patients (47.2% vs. 34.4% and 17.3% vs. 8.5% respectively).

### 3.2 Cost outcomes

The unadjusted mean total medical costs for the surgical and non‐surgical groups among members are reported in Table 3 while the adjusted cost differences are presented in Table 4. After adjustment, the surgery group had significantly lower total annual medical cost starting in Year 2 (cost difference = −$938, 95% CI: −$1165, −$706) after the surgery episode, which was sustained through Year 10 (cost difference = −$1241, 95% CI: −$2287, −$101) compared to the non‐surgical group. Unadjusted cost and adjusted cost differences in total healthcare cost (sum of medical and pharmacy cost) were also calculated among the subset of the population with both medical and pharmacy enrollment demonstrating similar results; see Table S2 for sample size and Table S3 for cost differences.

**TABLE 3:** Unadjusted total medical costs for surgical and non‐surgical groups by follow‐up period.

	Total medical expenditure^a^	
	Surgery, mean (sd)	Non‐surgery, mean (sd)
Operative	$28,580 (27,538)	$66 (1612)
Post 30‐day	$2321 (15,501)	$1133 (8038)
Year 1	$13,124 ($28,015)	$13,275 ($29,676)
Year 2	$12,494 ($28,028)	$13,312 ($31,474)
Year 3	$12,513 ($28,430)	$13,610 ($32,319)
Year 4	$12,629 ($28,949)	$14,479 ($33,939)
Year 5	$12,513 ($28,430)	$13,610 ($32,319)
Year 6	$12,982 ($29,797)	$15,608 ($35,804)
Year 7	$13,394 ($31,011)	$15,489 ($34,665)
Year 8	$13,688 ($31,562)	$15,639 ($34,844)
Year 9	$13,114 ($30,247)	$16,042 ($36,156)
Year 10	$13,761 ($32,830)	$16,226 ($34,675)

^a^ Among members with at least 1 day of medical eligibility in a given year. Total medical cost includes sum of inpatient, emergency and outpatient costs. High costs were capped at $250,000 to avoid results that were skewed by outlier cases. Costs were inflation‐adjusted to their 2019 value and annualized to account for partial year plan enrollments.

**TABLE 4:** Adjusted mean total medical cost differences for surgical and non‐surgical groups by follow‐up period.

	Cost difference^a^	95% CI	*p* Value
Year 1	$126	−$85, $342	0.2421
Year 2	−$938	−$1165, −$706	<0.0001
Year 3	−$1280	−$1551, −$1001	<0.0001
Year 4	−$1474	−$1737, −$1204	<0.0001
Year 5	−$1104	−$1338, −$861	<0.0001
Year 6	−$2275	−$2779, −$1752	<0.0001
Year 7	−$1370	−$1934, −$780	<0.0001
Year 8	−$1198	−$1827, −$533	0.0006
Year 9	−$1763	−$2474, −$1001	<0.0001
Year 10	−$1241	−$2287, −$101	0.0335

^a^Among members with at least 1 day of medical eligibility in given year. Patients with zero costs during a study year were assigned a cost of $1 and high costs were capped at $250,000 to avoid results that were skewed by outlier cases. Costs were inflation‐adjusted to their 2019 value and annualized to account for partial year plan enrollments.

**FIGURE 3:**
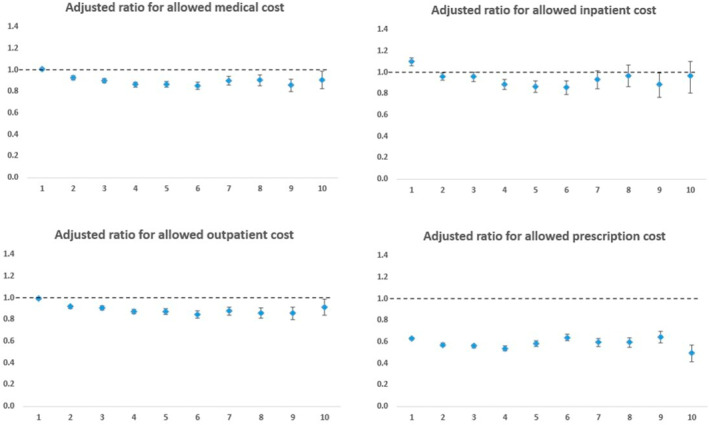
Adjusted cost ratios of the surgical group versus non‐surgical group by follow‐up year and cost category. Total medical cost includes sum of inpatient, emergency, and outpatient costs. Pharmacy cost ratios were calculated among a subset of members with pharmacy enrollment. High costs were capped at $250,000 and costs were inflation‐adjusted to their 2019 value.

Figure 3 shows the cost ratios of the surgical group compared to the non‐surgical group by cost categories and follow‐up periods, after adjusting for baseline demographic and clinical characteristics. The total medical cost (sum of INP, ER, and OUT) was lower from Year 2 (cost ratio = 0.93, 95% CI: 0.91, 0.95) to Year 10 (cost ratio = 0.91, 95% CI: 0.84, 0.99). Among the cost categories, the surgical group had lower INP cost from Year 2 (cost ratio = 0.96, 95% CI: 0.93, 0.997) to Year 6 (cost ratio = 0.86, 95% CI: 0.80, 0.93), except for Year 3; lower cost of OUT services from Year 2 (cost ratio = 0.92, 95% CI: 0.90, 0.94) to Year 10 (cost ratio = 0.92, 95% CI: 0.84, 0.995); and consistently lower pharmacy cost from Year 1 (cost ratio = 0.63, 95% CI: 0.61, 0.65) to Year 10 (cost ratio = 0.50, 95% CI: 0.43, 0.58), after surgery compared to the non‐surgical group. The results from the sensitivity analysis among members with 5 years of continuous medical and pharmacy enrollment were consistent with the main analysis. (see Table S4.)

### 3.3 SES analysis results in surgical cohort

**FIGURE 4:**
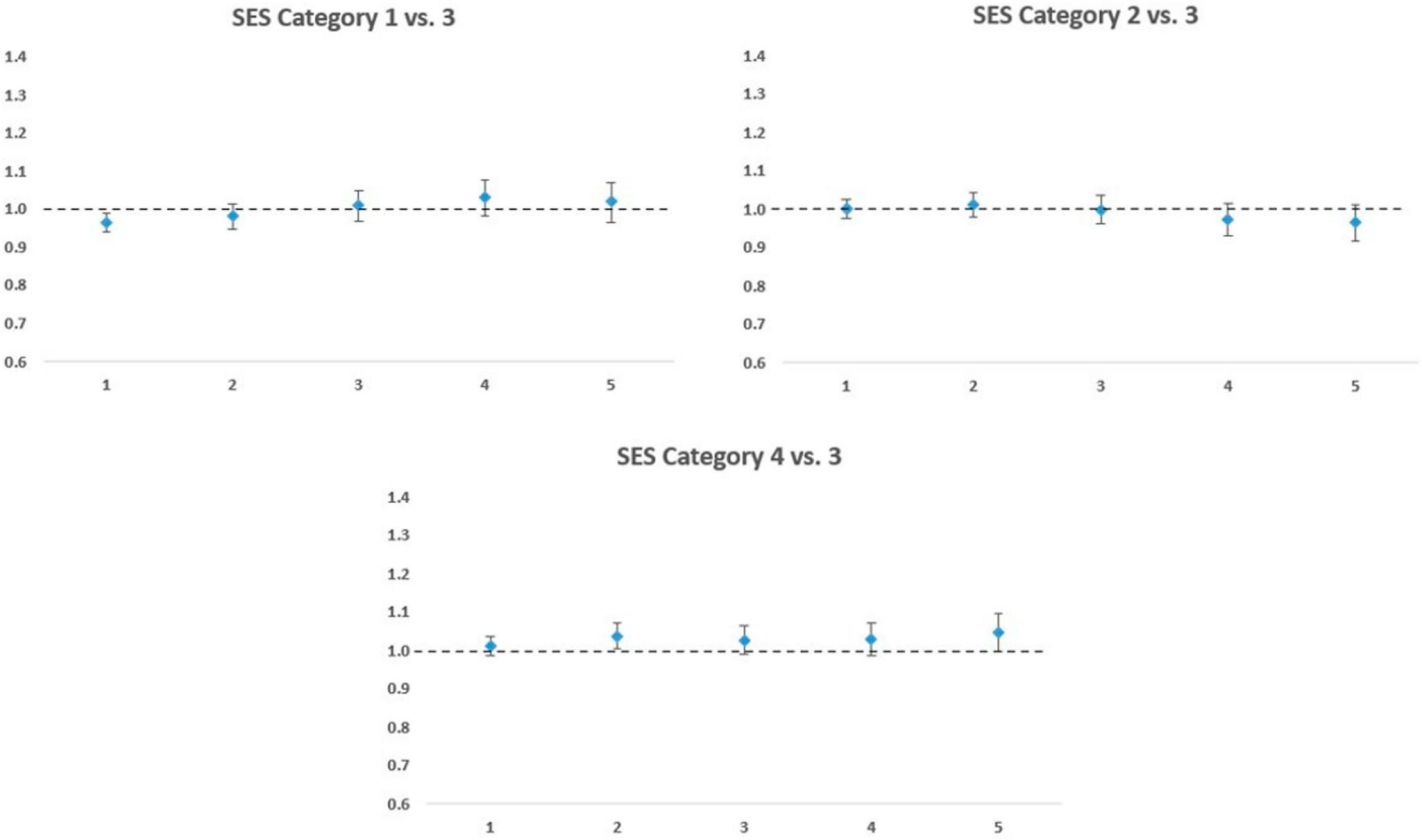
Adjusted cost ratios for total medical cost among the surgery group in the 5‐year follow‐up period by socio‐economic status quartiles. High costs were capped at $250,000 and costs were inflation‐adjusted to 2019 value.

Figure 4 shows the adjusted cost ratios along with confidence intervals in a 5 year follow‐up period of all SES quartiles compared to the 3^rd^ SES quartile as the reference. No significant cost differences were seen between the 1^st^ and 2^nd^ SES quartiles when compared with the 3^rd^ SES quartile. The 4^th^ SES quartile had slightly higher costs in Year 2 (cost ratio = 1.03, 95% CI: 1.01–1.07) compared to the 3^rd^ SES Quartile. The mean unadjusted cost of the surgery group stratified by SES category from year 1 to year 5 are reported in Table S5.

## 4 DISCUSSION

These findings show that overall total medical costs for individuals with severe obesity enrolled in commercial health plans who underwent BS were slightly lower or similar in cumulative post‐index years (Year 1–Year 10) compared with their counterparts who did not undergo BS. There were major pharmacy cost savings (i.e., reduced medication spending) in the surgery group through all 10 postoperative periods.

The findings of this study are more encouraging than prior studies where there have been little to no spending reductions following BS.^17^, ^18^ For example, the study by Weiner et al., using 2002–2008 data, reported that the surgical group had a higher overall cost during the second and third years following surgery and the cost was not different in the later years compared to non‐surgical group.^17^ Lower prescription and office visit costs were seen among the surgical group; however, they were offset by higher INP costs.^17^ In this study, BS was associated with reduced medical cost from Year 2 to Year 10, which could be due to advances in surgical procedures over the past years. Weiner's study consisted of patients who underwent BS from 2002 to 2008, in which 12.3% of study participants AGB, 38.3% had laparoscopic gastric bypass, and 35.4% had open gastric bypass. However, 46.8% of patients in the current study underwent the SG procedure, which has shown to be safer with less risk of hospitalization in both the short‐ and long‐term than AGB and bypass.^11^, ^13^

A study by Smith et al., that looked at bariatric surgeries between 2000 and 2011 using Veterans' Affairs (VA) health care system data to study 10‐year downstream healthcare cost, saw increases in total health care expenditures in the first 2 years after BS and then observed similar costs to the non‐surgery group in the next 8 years.^18^ In contrast, this study saw increased Year 1 cost followed by decreased Year 2 to Year 10 cost in the surgery group. The VA study also had an older population: mean age of 52 years in the surgery cohort compared to this study with a mean age of 45 years. In addition, it also had fewer surgery patients (15%) who underwent the SG procedure compared to this study.

Having information on outcomes by SES is relevant to the overall goal of helping individuals with weight management. This study examined downstream cost impact on the surgery group by patients' neighborhood‐level SES, testing the hypothesis that patients in lower SES groups may not have the same level of positive outcomes, due to environmental and social factors such as access to healthy food (e.g., living in a food desert) and/or income constraints (driving food insecurity). However, no major cost differences associated with patients' SES were observed when they were compared based on four SES quartiles up to 5 years after the surgery. To the authors' knowledge, this is the first study using commercial insurance data to examine the follow‐up cost post‐surgery by members' SES. Prior studies focused on the association between weight loss and SES status and the results have been mixed.^16^, ^20^, ^27^, ^28^ One of the studies examined the association between individual‐ and neighborhood‐level sociodemographic factors and surgical weight loss at 1 and 3 years and found no significant association between census tract‐level Neighborhood Deprivation Index score and weight loss at either time point.^28^ This study's findings of similar outcomes by SES may be due to the advances in the procedures and the robustness of the sample. This is an important finding given that there has, in recent years, been an increase in the use of the procedure among lower income individuals.^29^

This large cohort study used recent claims data to identify BS cases and examined their long‐term cost impact. The study features a broad group of the commercially insured population, a wide range of bariatric surgical procedures including a high proportion with SG, and a long follow‐up time. Matching patient characteristics, particularly BMI diagnosis, improves the validity of the study.

5

A concern could arise regarding the non‐surgical group in the study as they did not have any bariatric procedure during the baseline and follow‐up periods. Using a future event, “no bariatric procedure,” to define the non‐users could induce bias, as people with BS are different from the people who never it. Of note, several tactics were applied to address this issue. First, an index date was created for the non‐surgical group in a way that approximated the disease progression and timing for surgery of the surgery patients. Second, exact matching was performed on key demographic and clinical characteristics, most importantly on the BMI category codes, to ensure that the two groups were similar. Recent studies have shown that obesity‐related ICD codes can accurately identify patients with obesity using healthcare claims databases with one of the studies reporting specificity >97% for all categories of BMI.^16^, ^17^ In the regression analysis, there was an adjustment for wide‐ranging baseline factors. Finally, while there may be remaining unmeasured variables that may cause the surgical and non‐surgical groups to be non‐equivalent, such as information about lifestyle, diet, and exercise pattern, *E*‐values were analyzed (see Table S6), and all the *E*‐values ranged 1.06–2.19. Cost ratios were also calculated for other measured confounders (see Table S7) for total medical cost for year one and they ranged 0.4–1.25; the mean was 0.94, which is smaller in absolute value than the *E*‐value for year 1 total medical cost, suggesting cautious optimism that unmeasured confounding would likely not substantially change the results of the analyses.

ICD 10 diagnostic codes for BMI categories were better documented in claims in 2015 and beyond. Yet, the analysis adjusted for the year of index date to try to account for changes in documentation over time. Also, for the main analysis, caution is needed in interpreting results in later follow‐up years as sample sizes dropped, especially in the non‐surgery group. Another limitation is that the baseline characteristics and the members' SES status were measured prior to the surgery or on the date of surgery and they might have less impact on the outcomes that occurred a long time after the surgery. For the secondary analysis by SES status, 5 years of data instead of 10 were analyzed to ensure a comparable sample size in each quartile in follow‐up years. Finally, this study was conducted on a commercially insured population. Therefore, the economic findings might not be generalized to other populations in different insurance plans, such as Medicaid.

## 6 CONCLUSION

This study found reductions in annual medical health care costs for up to 10 years post‐BS. Furthermore, among patients who underwent BS, patients living in neighborhoods with differing levels of SES had similar cost reductions. The shift to safer and less invasive bariatric procedures likely contributed to the greater levels of cost reductions observed in this study. These results can help policy makers and employers to design coverage for bariatric procedures, and to prioritize the surgery options offering the best long‐term value.

## AUTHOR CONTRIBUTIONS

**Sonali Shambhu**: Methodology; investigation; analysis; interpretation of data; original draft preparation. **Qinli Ma**: Conceptualization; methodology; investigation; interpretation of data. **Aliza S. Gordon**: Supervision; investigation; interpretation of data; writing – reviewing and editing. **David Pryor**: Supervision; funding acquisition. **Joseph A. Karam:** Supervision; funding acquisition. **Andrea DeVries**: Conceptualization; methodology; supervision; interpretation of data; original draft preparation. All authors read and approved the final manuscript.

## CONFLICT OF INTEREST STATEMENT

Sonali Shambhu, Aliza S. Gordon, David Pryor, and Joseph A. Karam hold Elevance Health (ELV) stocks. All other authors have no conflicts of interest.

## Supporting information

Supporting Information S1

## Data Availability

The datasets generated and/or analyzed during the current study are not publicly available due to the reason that the raw data are protected and are not available due to data privacy laws. Investigators with an academic affiliation may contact the corresponding author for data access for the purposes of validating the above findings. Requests will be processed within 60 days.
