# Supplementary material for: Long term cost outcomes among commercially insured patients undergoing bariatric surgical procedures
Source: Obes Sci Pract. 2024 Jan 1;10(1):e727. doi: 10.1002/osp4.727 (PMC10766043; doi:10.1002/osp4.727)
Supplement: Supplementary file 1 — Supporting Information S1 [file OSP4-10-e727-s001.docx]

**TITLE:** Long Term Cost Outcomes Among Commercially Insured Patients Undergoing Bariatric Procedures

**AUTHORS:** Sonali Shambhu, Qinli Ma, Aliza Gordon*, David Pryor, Joseph A. Karam, Andrea DeVries

**AFFILIATION**Sonali Shambhu, MPH, Sonali.shambhu@elevancehealth.com, Public Policy Institute, Elevance Health, Indianapolis, IN

Qinli Ma, PhD, qma@healthcore.com, Enterprise Health Service Research, Anthem, Wilmington, DE

Aliza Gordon*, PhD, aliza.gordon@elevancehealth.com, Public Policy Institute, Elevance Health, Indianapolis, IN

David Pryor, MD, david.pryor@anthem.com, Clinical Strategy and Innovation, Anthem, Woodland Hills, CA

Joseph A. Karam, MD, joseph.karam@anthem.com, Grievances & Appeals, Anthem, Woodland Hills, CA

Andrea DeVries, PhD, Andrea.DeVries@elevancehealth.com, Public Policy Institute, Elevance Health, Indianapolis, IN

**CONTACT INFO:**

Aliza Gordon

220 Virginia Avenue

Indianapolis, IN 46204

Phone:(800) 331-1476 ; Email: aliza.gordon@elevancehealth.com

**Table of Contents**

| Table S1 | ICD 9/10 BMI codes mapped to BMI categories in Study |
| --- | --- |
| Table S2 | Number of patients in study group during 10 follow-up periods among members with medical and pharmacy eligibility |
| Table S3 | Unadjusted Total Healthcare Costs (Medical +Pharmacy) and Adjusted Cost Differences for Surgical and Non-Surgical Groups among members with at least 1 day of Medical and Pharmacy enrollment in 10 Follow-up Periods. |
| Table S4 | Unadjusted Total Medical and Healthcare Costs (Medical +Pharmacy) for Surgical and Non-Surgical Groups among members with 5 year continuous Medical and Pharmacy benefits by Follow-up Periods |
| Table S5 | Total Medical unadjusted cost by SES category from year 1 to 5 post bariatric surgery. |
| Table S6 | E- values for all cost ratios results in Figure 3 |
| Table S7 | Cost ratios for all measured confounders for year 1 total medical cost. |

**Table S1**: ICD 9/10 BMI codes mapped to BMI categories in Study

| ICD 10 codes | Description |
| --- | --- |
| E66.01 | Morbid (severe) obesity due to excess calories |
| E66.09 | Other obesity due to excess calories |
| E66.1 | Drug-induced obesity |
| E66.2 | Morbid (severe) obesity with alveolar hypoventilation |
| E66.8 | Other obesity |
| E66.9 | Obesity, unspecified |
| Z68.30 | Body mass index (BMI) 30.0-30.9, adult |
| Z68.31 | Body mass index (BMI) 31.0-31.9, adult |
| Z68.32 | Body mass index (BMI) 32.0-32.9, adult |
| Z68.33 | Body mass index (BMI) 33.0-33.9, adult |
| Z68.34 | Body mass index (BMI) 34.0-34.9, adult |
| Z68.35 | Body mass index (BMI) 35.0-35.9, adult |
| Z68.36 | Body mass index (BMI) 36.0-36.9, adult |
| Z68.37 | Body mass index (BMI) 37.0-37.9, adult |
| Z68.38 | Body mass index (BMI) 38.0-38.9, adult |
| Z68.39 | Body mass index (BMI) 39.0-39.9, adult |
| ICD 9 codes | Description |
| V85.4 | BMI 40 and over, adult |
| V85.30 | Body Mass Index 30.0-30.9, adult |
| V85.31 | Body Mass Index 31.0-31.9, adult |
| V85.32 | Body Mass Index 32.0-32.9, adult |
| V85.33 | Body Mass Index 33.0-33.9, adult |
| V85.34 | Body Mass Index 34.0-34.9, adult |
| V85.35 | Body Mass Index 35.0-35.9, adult |
| V85.36 | Body Mass Index 36.0-36.9, adult |
| V85.37 | Body Mass Index 37.0-37.9, adult |
| V85.38 | Body Mass Index 38.0-38.9, adult |
| V85.39 | Body Mass Index 39.0-39.9, adult |
| V85.41 | Body Mass Index 40.0-44.9, adult |
| V85.42 | Body Mass Index 45.0-49.9, adult |
| V85.43 | Body Mass Index 50.0-59.9, adult |
| V85.44 | Body Mass Index 60.0-69.9, adult |
| V85.45 | Body Mass Index 70 and over, adult |
| 278.01 | Morbid obesity |
| 278.03 | Obesity hypoventilation syndrome |
| 278.00 | Obesity, unspecified |
| Z68.41 | Body mass index (BMI) 40.0-44.9, adult |
| Z68.42 | Body mass index (BMI) 45.0-49.9, adult |
| Z68.43 | Body mass index (BMI) 50.0-59.9, adult |

**Table S2:** Number of patients in study group during 10 follow-up periods among members with medical and pharmacy eligibility

| **Study group** | **Year 1** | **Year 2** | **Year 3** | **Year 4** | **Year 5** | **Year 6** | **Year 7** | **Year 8** | **Year 9** | **Year 10** |
| --- | --- | --- | --- | --- | --- | --- | --- | --- | --- | --- |
| **Non-Surgical Group** | 36,127 | 29,834 | 19,371 | 12,494 | 8,455 | 5,765 | 3,989 | 2,732 | 1,697 | 1,020 |
| **Surgical Group** | 27,852 | 23,404 | 17,556 | 13,765 | 11,097 | 8,885 | 6,734 | 5,065 | 3,635 | 2,342 |

**Table S3:** Unadjusted Total Healthcare Costs (Medical +Pharmacy) and adjusted cost differences for Surgical and Non-Surgical Groups among members with at least 1 day of Medical and Pharmacy enrollment in 10 Follow-up Periods.

| **Total Healthcare Cost* (Med+RX)** | **Unadjusted cost** | | **Adjusted Cost differences** | | |
| --- | --- | --- | --- | --- | --- |
|  | **Surgery (mean/sd)** | **Non-Surgery (mean/sd)** | **Cost Difference** | **95% CI** | **P value** |
| **Year 1** | $18,290 ($34,643) | $19,568 ($35,015) | -850 | ($1,256), ($434) | <.0001 |
| **Year 2** | $17,189 ($34,099) | $20,087 ($37,652) | -3358 | ($3,839), ($2,861) | <.0001 |
| **Year 3** | $17,322 ($34,245) | $20,570 ($38,251) | -3327 | ($3,865), ($2,769) | <.0001 |
| **Year 4** | $17,093 ($34,062) | $21,614 ($39,431) | -3783 | ($4,319), ($3,225) | <.0001 |
| **Year 5** | $17,322 ($34,245) | $20,570 ($38,251) | -3046 | ($3,631), ($2,432) | <.0001 |
| **Year 6** | $17,753 ($35,134) | $21,725 ($38,939) | -3467 | ($4,480), ($2,392) | <.0001 |
| **Year 7** | $17,603 ($35,018) | $22,291 ($37,788) | -3180 | ($4,193), ($2,097) | <.0001 |
| **Year 8** | $18,072 ($35,400) | $22,926 ($39,277) | -2073 | ($2,848), ($1,233) | <.0001 |
| **Year 9** | $17,442 ($34,146) | $23,864 ($40,803) | -2537 | ($3,624), ($1,337) | <.0001 |
| **Year 10** | $17,493 ($36,367) | $25,528 ($41,642) | -4372 | ($5,691), ($2,880) | <.0001 |

***** Among members with at least 1 day of medical and pharmacy eligibility in given year. Patients with zero costs during a study year were assigned a cost of $1 and high costs were capped at $250 000 to avoid results that were skewed by outlier cases. Costs are inflation-adjusted to the 2019 value and annualized to account for partial year plan enrollments.

**Table S4**: Unadjusted Total Medical and Total Healthcare Costs for Surgical and Non-Surgical Groups among members with 5 year continuous Medical and Pharmacy benefits by Follow-up Periods (Sensitivity Analysis)

| **Total medical cost* (INP, ER, OUT)** | **Surgery (n=23,285)** | | **Non-Surgery (n=10,745)** | | **Total Healthcare cost** (medical + pharmacy)** | **Surgery (n=5,577)** | | **Non-Surgery (n=3,840)** | |
| --- | --- | --- | --- | --- | --- | --- | --- | --- | --- |
|  | **Mean** | **Std** | **Mean** | **Std** |  | **Mean** | **Std** | **Mean** | **Std** |
| Year 1 | $12,867 | $35,711 | $13,717 | $33,183 | Year 1 | $15,963 | $35,282 | $17,378 | $31,040 |
| Year 2 | $12,022 | $31,150 | $14,190 | $38,079 | Year 2 | $15,180 | $30,999 | $18,464 | $35,420 |
| Year 3 | $12,314 | $30,098 | $14,843 | $39,316 | Year 3 | $15,927 | $32,908 | $20,268 | $42,809 |
| Year 4 | $12,832 | $39,692 | $15,433 | $41,340 | Year 4 | $16,381 | $36,166 | $20,553 | $36,951 |
| Year 5 | $13,774 | $37,236 | $16,344 | $43,416 | Year 5 | $18,020 | $42,423 | $22,362 | $41,200 |

***** Among members with at least 1 day of medical eligibility in given year. ****** Among members with at least 1 day of medical and pharmacy eligibility in given year. Patients with zero costs during a study year were assigned a cost of $1 and high costs were capped at $250 000 to avoid results that were skewed by outlier cases. Costs are inflation-adjusted to the 2019 value and annualized to account for partial year plan enrollments.

**Table S5:** Total Medical unadjusted cost by SES category from year 1 to 5 post bariatric surgery.

|  | **SES category 1** | **SES category 2** | **SES category 3** | **SES category 4** |
| --- | --- | --- | --- | --- |
| Baseline | $12,801 | $13,459 | $13,471 | $14,399 |
| Year1 | $12,168 | $12,804 | $12,876 | $13,217 |
| Year2 | $10,885 | $11,302 | $11,450 | $11,993 |
| Year3 | $9,714 | $10,032 | $10,187 | $10,664 |
| Year4 | $9,653 | $9,538 | $9,862 | $10,423 |
| Year5 | $9,156 | $9,374 | $9,866 | $10,484 |

**Table S6:** E- values for all cost ratios results in Figure 3

|  | **E-values Total medical cost ratios** | **E-values INP cost ratios** | **E-values OUT cost ratios** | **E-values Rx cost ratios** |
| --- | --- | --- | --- | --- |
| **Year 1** | 1.071802 | 1.275649 | 1.056052 | 1.829749 |
| **Year 2** | 1.237599 | 1.162975 | 1.251967 | 1.975745 |
| **Year 3** | 1.28769 | 1.168278 | 1.280115 | 2.006226 |
| **Year 4** | 1.356863 | 1.313004 | 1.342986 | 2.065878 |
| **Year 5** | 1.353906 | 1.352467 | 1.339866 | 1.947511 |
| **Year 6** | 1.383302 | 1.370512 | 1.393515 | 1.810354 |
| **Year 7** | 1.292734 | 1.226546 | 1.335111 | 1.919008 |
| **Year 8** | 1.2784 | 1.136221 | 1.367273 | 1.913398 |
| **Year 9** | 1.370481 | 1.312544 | 1.368693 | 1.794943 |
| **Year 10** | 1.272239 | 1.149182 | 1.25981 | 2.190144 |

**Table S7: Cost ratios for all measured confounders for year 1 total medical cost**.

| Parameter | Cost Ratios |
| --- | --- |
| Age | 1.01 |
| Male | 0.95 |
| Female | 1.00 |
| obesityBMIMatchingCat1 | 0.55 |
| obesityBMIMatchingCat2 | 0.49 |
| obesityBMIMatchingCat3 | 0.47 |
| obesityBMIMatchingCat4 | 0.46 |
| obesityBMIMatchingCat5 | 0.45 |
| obesityBMIMatchingCat6 | 0.45 |
| obesityBMIMatchingCat7 | 0.93 |
| obesityBMIMatchingCat8 | 1.00 |
| bLACInpatientVisitCnt | 1.13 |
| baseECI | 1.15 |
| Region_cat2 | 1.04 |
| Region_cat3 | 0.96 |
| Region_cat4 | 1.06 |
| Region_cat5 | 1.16 |
| Region_cat1 | 1.00 |
| healthPlanType - CDHP | 1.03 |
| healthPlanType - HMO | 1.00 |
| healthPlanType - Unknown | 0.86 |
| healthPlanType - PPO | 1.00 |
| Base_MedRx_flg | 0.98 |
| sESIndexCategory 2 | 0.99 |
| sESIndexCategory 3 | 1.01 |
| sESIndexCategory 4 | 1.02 |
| sESIndexCategory 5 | 1.00 |
| sESIndexCategory 1 | 1.00 |
| indexYear2 | 0.97 |
| indexYear3 | 0.96 |
| indexYear4 | 0.92 |
| indexYear5 | 0.92 |
| indexYear6 | 0.90 |
| indexYear7 | 0.89 |
| indexYear8 | 0.82 |
| indexYear9 | 0.79 |
| indexYear10 | 0.78 |
| indexYear1 | 1.00 |
| BaseM6_12ER | 1.22 |
| BaseM6_12cost | 1.00 |
| Baseline Hyperlipidemia | 0.95 |
| Baseline Diabetes Mellitus | 0.93 |
| Baseline Gall Bladder | 1.10 |
| Baseline Sleep Apnea | 1.07 |
| Baseline Metabolic Syndrome | 0.96 |
| Baseline Anxiety | 1.13 |
| Baseline Depression | 1.01 |
| Baseline GERD | 1.05 |
| Baseline Hypertension | 0.90 |
| Baseline CKD | 1.09 |
| Baseline NaFLD | 0.95 |
| Baseline Osteoarthritis | 1.25 |
| Baseline Thrombosis | 1.19 |
| Baseline Eating Disorder | 1.01 |
